# CT energy spectral parameters of creeping fat in Crohn’s disease and correlation with inflammatory activity

**DOI:** 10.1186/s13244-023-01592-6

**Published:** 2024-01-17

**Authors:** Xianchu Li, Wei Wu, Yan Yuan, Zhiming Zhu, Xiaowei Liu, Desheng Xiao, Xueying Long

**Affiliations:** 1 Department of Radiology, The Second Affiliated Hospital, Hengyang Medical School, University of South China, Hengyang, China; 2 Department of Radiology, Xiangya Hospital, Central South University, 87 Xiangya Road, Changsha, 410008 China; 3 Department of General Surgery, Xiangya Hospital, Central South University, Changsha, China; 4 Department of Gastroenterology, Xiangya Hospital, Central South University, Changsha, China; 5 Department of Pathology, Xiangya Hospital, Central South University, Changsha, China; 6 National Clinical Research Center for Geriatric Disorders, Xiangya Hospital, Central South University, Changsha, China

**Keywords:** Creeping fat, Crohn’s disease, CT enterography, Dual-energy, Mesenteric adipose tissue

## Abstract

**Objectives:**

Creeping fat is a kind of unique abnormal mesenteric tissue at the sites of diseased bowel of Crohn’s disease. By using dual-energy CT enterography, this study aimed to evaluate the feasibility of spectral parameters in the quantitative analysis of mesenteric adipose tissue or creeping fat.

**Methods:**

In this study, patients with known or suspected Crohn’s disease who underwent dual-energy CT enterography from March 1, 2019, to March 31, 2021, were enrolled. Among them, 40 patients with surgery and pathology-proven creeping fat were selected as the creeping fat Crohn’s disease group, and 40 normal patients were selected as the control group. The quantitative spectral parameters including the slope of the Hounsfield unit curve, normalised fat–water concentration, normalised fat-iodine concentration, and normalised fat volume fraction at the enteric phases were obtained. Mann–Whitney *U* test, Kruskal–Wallis *H* test, and receiver operating characteristic curve analysis were applied to compare quantitative parameters among various groups.

**Results:**

A significant difference was observed in the slope of the Hounsfield unit curve, normalised fat–water concentration, normalised fat-iodine concentration, and normalised fat volume fraction between mesenteric adipose tissue and creeping fat with Crohn’s disease at the enteric phase (all* p* < 0.001). The slope of the Hounsfield unit curve of creeping fat at the enteric phase had a better capability to distinguish inactive and active Crohn’s disease (AUC = 0.93, *p* < 0.001).

**Conclusion:**

Dual-energy CT enterography with quantitative spectral parameters is a potentially novel noninvasive tool for evaluating creeping fat in Crohn’s disease.

**Critical relevance statement:**

Energy spectral parameters of creeping fat in Crohn’s disease are significantly different from normal mesenteric adipose tissues and are correlated with inflammatory activity.

**Key points:**

• Dual-energy CT enterography allows quantitatively assessing creeping fat with spectral parameters.

• The creeping fat has distinct spectral parameters to normal mesenteric adipose.

• The spectral parameters accurately differentiate active and inactive Crohn’s disease.

**Graphical Abstract:**

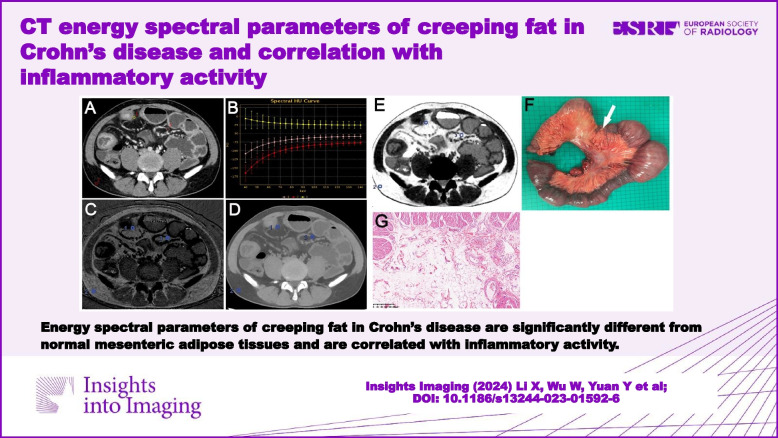

## Introduction

Recent studies have shown that the mesentery, a newly recognised independent organ [1, 2], interacts closely with the affected bowel and plays a potential role in the development process of Crohn’s disease (CD) [3].

Creeping fat, also known as “wrapping fat”, which was first described by Crohn et al. [4], is a kind of abnormal mesenteric adipose tissue (MAT) characterised by surrounding > 50% of the intestinal circumference of affected bowel in CD [5]. Creeping fat is seen not in ulcerative colitis (another form of inflammatory bowel disease) but only in CD [6]. Histopathological analysis shows that creeping fat is a low-grade, chronic inflammatory process of MAT [7]. Creeping fat exhibits hyperplasia with increased small round adipocytes, accompanied by vascular proliferation, fibrosis, lymphadenectasis, and stromal cell infiltration [8]. It is often regarded as an anatomic landmark for the resection of affected bowel segments by surgeons during operation [9–13] and is associated with fibrosis, stricturing complications, and surgical recurrence in CD [3, 5, 6, 14–16].

Owing to a rapid scan speed and excellent image quality in the era of multi-detector row CT, CT enterography (CTE) is an appropriate imaging modality for the evaluation of CD. Although the value of CTE in quantitatively assessing mural inflammation in CD is well recognised [17–19], Sakurai et al. [20] found that the mesenteric findings of CTE, rather than the mural findings, were highly correlated with the endoscopically evaluated severity of ulceration. Similarly, Feng et al. [21] found that creeping fat was closely related to inflammation activity under endoscopy.

Dual-energy CT provides enhanced diagnostic power with similar or even reduced radiation dose as compared to single-energy CT [22–24]. Dual-energy CTE with material density images and virtually monochromatic images can distinguish different tissues according to the behaviours of the material at different energy levels [23]. These spectral parameters may help quantitatively assess mesenteric tissues in CD. However, the spectral parameters of creeping fat correlated with the surgical and pathologic findings of CD have not been reported so far. By using a series of quantitative dual-energy parameters, this study aimed to quantitatively evaluate the differences between MAT in healthy controls and creeping fat in CD patients and investigate the correlations with inflammatory activities in CD patients.

## Materials and methods

### Ethical *considerations*

Our prospective, single-centre study was approved by the Medical Ethics Committee of the Xiangya Hospital of Central South University. The written informed consent for the dual-energy CTE scan was obtained from all participants.

### Study participants

The inclusion criteria were as follows: (a) patients with a confirmed diagnosis by endoscopy, histopathology, clinical features, diagnostic imaging, and laboratory findings [25] and (b) patients without contraindications or previous adverse reactions to iodine contrast media and who could successfully finish dual-energy CTE examination. The exclusion criteria were described below: (a) patients with the preparation of intestinal tract for dual-energy CTE scanning but who were scanned in dual-energy CT angiography and CT venography of superior mesenteric artery and vein (to exclude mesenteric thrombotic diseases); (b) patients with too poor image quality of dual-energy CTE and serious artefacts affecting the measurement of creeping fat; (c) patients whose dual-energy CTE examination was not standardised (e.g. the portal phase was scanned, instead of the enteric phase).

**Fig. 1:**
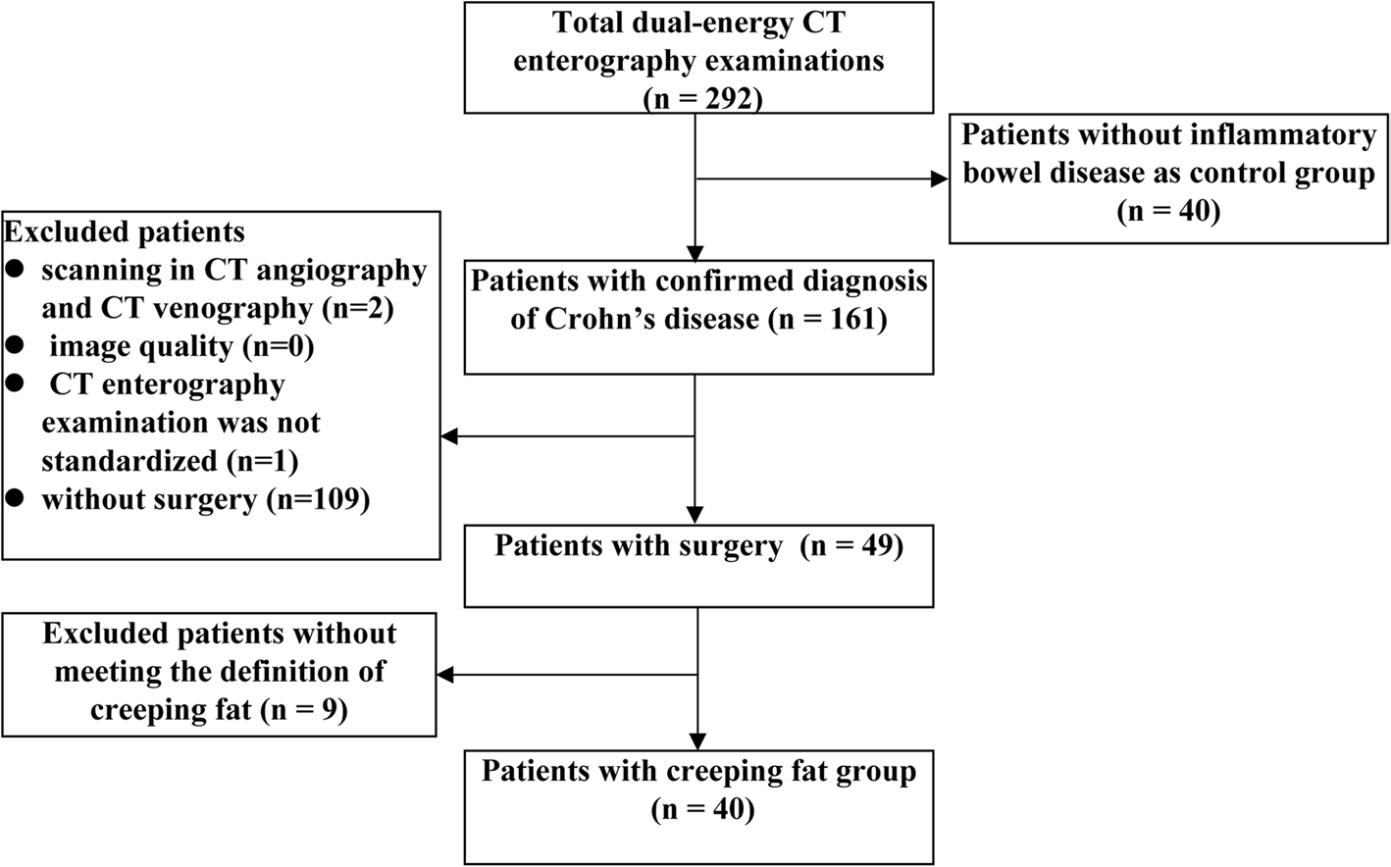
Flowchart of study enrollment

Totally, 292 dual-energy CTE scans were performed from March 1, 2019, to March 31, 2021, in our hospital, including the scans for 161 patients with a confirmed diagnosis of CD. In these 161 CD patients, 3 cases were excluded according to the exclusion criteria, 109 cases were treated with medical drugs, and 49 cases underwent subsequent surgery for CD complications (intestinal obstruction, abdominal abscess, and fistula formation) and medical treatment failure [26]. According to the intraoperative exploration findings and the postoperative pathologic results, 40 cases of CD with creeping fat were selected as the creeping fat group and 9 cases of CD whose MAT covered < 50% of the affected bowel circumference [5] were excluded because such coverage did not meet the standard of creeping fat. Meanwhile, 40 cases of non-CD patients with normal MAT were selected as the control group. The detailed inclusion criteria of the control group were as follows: (a) patients with various symptoms such as abdominal distention, abdominal discomfort, diarrhoea, and abdominal pain, and received dual-energy CTE to screen bowel disease; (b) no obvious abnormalities were found in the abdomen except for small hepatic cysts, renal cysts, ovarian cysts, noncomplicated gallstone, small renal calculus, and small myoma of the uterus (these abnormal findings were considered as not clinically significant); (c) without obvious gastrointestinal tract diseases, such as inflammatory bowel disease, intestinal tuberculosis, gastrointestinal cancer, and intestinal Behcet’s disease, which were carefully confirmed by a comprehensive diagnosis of clinical features, endoscopy, radiological imaging, and laboratory findings (Fig. 1).

### Dual-energy CTE scanning protocol

All patients underwent dual-energy CTE scanning on a 256-detector row CT scanner (Revolution CT, GE Healthcare, USA) using a uniform protocol. The patients were asked to have a low-residue diet during the day before the examination and keep fasting for over 10 h before the dual-energy CTE examination. One hour before scanning, the patients were required to drink a 2.5% mannitol solution (a total volume of 1200 to 1500 mL solution or more) at 20-min intervals (60, 40, and 20 min before scanning). Iopamidol injection (1.5 mL/kg body weight) was injected at a flow rate of 3 mL/s via the elbow vein using a power injector system. Contrast-enhanced CT scans were performed under the gemstone spectral imaging (GSI) scanning mode (with tube voltage fast switching between 80 and 140 kVp) at the enteric phase [27]. By utilising the bolus tracking technique, an individualised delay time for the enteric phase was determined as 30 s after the aorta reached the threshold of 100 HU. The scanning range was the full abdomen–pelvis from the diaphragmatic apex to the perineal bottom. The scanning parameters were as follows: tube current, 405 mA; scan type, helical; thickness, 1.25 mm; ASiR-V (adaptive statistical iterative reconstruction V) at 30% blending ratio; rotation time, 0.5 s; helical pitch, 0.984. Owing to the new-generation model-based iterative reconstruction ASIR-V, images acquired via this scanning protocol achieved an acceptable noise level and maintained diagnostic confidence at a low-radiation dose.

### Energy spectral reconstructions

All data of the dual-energy CT scans of the enteric phase were transferred to and analysed on the AW 4.7 workstation (GE Healthcare, USA) with GSI software.

On the workstation, the fat–water material basis images, fat-iodine material basis images, and virtual monochromatic (VMC) images with energies ranging from 40 to 100 keV were obtained (these spectral reconstructions were automatically done in 3–5 min per scan). Multi-material decomposition (MMD) algorithm [28, 29] was also applied in this study. Fat volume fraction (FVF) maps were generated using GSI Liver Fat software (AW 4.7 workstation; GE Healthcare, USA).

### Clinical *characteristics* of CD

The relevant clinical data of all patients were obtained from our institutional electronic medical record system. These records were collected when the patient was hospitalised. The Crohn’s disease activity index (CDAI) and Montreal classification of each CD patient were recorded. Inactive and active CD was defined as CDAI < 150 and CDAI ≥ 150, respectively [30].

### Image analysis and measurements

Creeping fat was defined as MAT covering more than half of the affected bowel circumference. In the prospective cohort, the imaging and surgical correlated evaluation of each patient was conducted by a radiologist with 20 years of experience in abdominal imaging and a surgeon with 15 years of experience in the surgical treatment of CD. The location and extent of the bowel lesion and the creeping fat on the preoperative CTE images were carefully assessed and matched with the surgical findings and pathological results of the resection specimens [16].

Two radiologists independently drew the ROIs and performed the measurements on the axial images. For CD patients, the ROIs of creeping fat were placed on the diseased mesentery with radiologic-surgical-pathological correlations. Moreover, the measurements on subcutaneous fat and normal mesentery were also conducted for each patient. The ROIs of creeping fat should be close to the affected bowel and avoid blood vessels and lymph nodes. Depending on the extent of creeping fat, the area of ROIs ranged from 7 to 30 mm^2^. The ROIs of subcutaneous fat were placed on the subcutaneous adipose tissue, avoiding subcutaneous muscles or possible subcutaneous lesions. The ROIs of normal mesentery were placed on the normal mesentery close to normal ileal bowels, avoiding blood vessels and lymph nodes. The shapes of ROIs were circular or elliptical. Once the ROIs were drawn, quantitative spectral parameters were automatedly shown. For the measurements of each tissue, three ROIs at different sites were selected and their means were calculated and recorded for each patient.

**Fig. 2:**
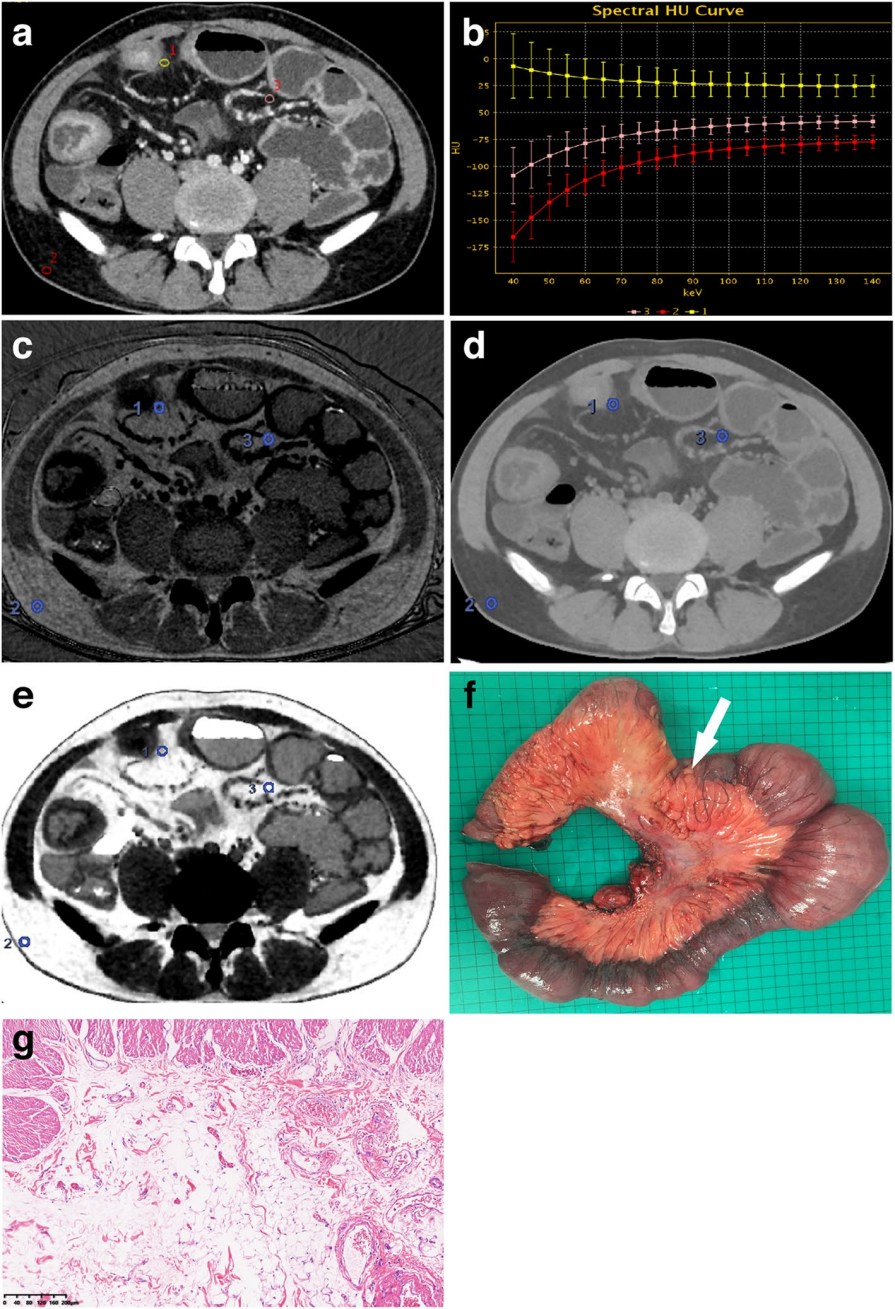
A 30-year-old male patient with inactive Crohn’s disease and non-stricturing and non-penetrating behaviours (**a**–**g**). The enteric phase CT enterography axial 70 keV virtual monochromatic (VMC) image (**a**) demonstrated the thickening of the ileal bowel wall as well as creeping fat, and the ROIs were selected in the creeping fat (ROI 1), the normal subcutaneous fat (ROI 2), and the “normal” mesenteric adipose tissue (ROI 3). **b** showed that compared with the subcutaneous fat (ROI 2) and “normal” mesenteric adipose tissue (ROI 3), the λ_HU_ curve of creeping fat (ROI 1) was inverted. **c**, **d**, and **e** showed fat–water material basis images, fat-iodine material basis images, and fat volume fraction maps at the enteric phase, respectively. The resected specimen (**f**) showed creeping fat wrapping around the affected ileal bowel loop. Pathological image (**g**) (HE, × 100) showed hyperplasia with increased small round adipocytes, vascular proliferation, and stromal cell infiltration in creeping fat

The normalised fat–water concentration (NFWC) was defined as the value of the fat–water concentration of the tissue divided by the subcutaneous fat–water concentration on fat–water material basis images. Similarly, the normalised fat-iodine concentration (NFIC) was defined as the value of the fat-iodine concentration of the tissue divided by the subcutaneous fat-iodine concentration on fat-iodine material basis images. The normalised FVF (NFVF) was defined as the value of the fat volume fraction of the tissue divided by the subcutaneous fat volume fraction on fat volume fraction images. The purpose of normalisation was to minimise individual differences. Approximately, the time for acquiring quantitative spectral parameters was 5–8 min per patient for a radiologist with preliminary experience. The slope of Hounsfield unit curves between 40 and 100 keV (λ_HU_) was calculated as the following equation: λ_HU_ = (CT attenuation of ROI on 40 keV VMC images − CT attenuation of ROI on 100 keV VMC images)/60. An example of the selected ROIs for measurements and calculations of λ_HU_, NFIC, NFWC, and NFVF of a CD patient with creeping fat is shown in Fig. 2.

### Statistical analysis

SPSS version 22.0 statistical software (IBM, Armonk, NY, USA) was used for statistical analysis. The Kolmogorov–Smirnov test was applied to evaluate whether the continuous variables had a normal distribution. The continuous variables were expressed as mean ± standard deviation (SD) or interquartile range (IQR), while the categorical variables were presented as number (*n*) and percentage (%). The continuous variables (λ_HU_, NFIC, NFWC, and NFVF at the enteric phase) had skewed distribution or heterogeneity of variance. Mann–Whitney *U* test was applied to compare the quantitative parameters of creeping fat with inflammatory activity at the enteric phase. Kruskal–Wallis *H* test was used for comparisons among MAT in the controls, “normal” MAT, and creeping fat in CD groups. Receiver operating characteristic (ROC) curve analysis was performed to evaluate the performance of quantitative parameters in assessing inflammatory activity of creeping fat. Then, the area under the curve (AUC), sensitivity, and specificity were separately calculated. A two-sided *p* value < 0.05 indicated a statistically significant difference.

## Results

### Participant characteristics

There were 40 creeping fat CD patients and 40 normal control patients included in this study. The clinical characteristics of 40 creeping fat CD patients are listed in Table 1. The patients with creeping fat CD were subdivided into the active group (CDAI ≥ 150) and the inactive group (CDAI < 150). In the creeping fat group, there were 26 males and 14 females, with an average age of 35. 58 ± 1.67 (16–60) years, and body mass index (BMI) of 18.22 ± 0.41 (14.31–24.65) kg/m^2^. Among CD patients, 60.0% (24 of 40) patients had ileal disease, 10.0% (4 of 40) patients had colonic disease, and 30.0% (12 of 40) patients had ileocolonic disease.

**Table 1:** Clinical characteristics of patients with Crohn’s disease according to Montreal classification

Clinical Characteristics	
Patient, *n* (%)	40
Sex, *n* (male/female)	(26/14)
Age, mean ± SD, years (range)	35.58 ± 1.67 (16–60)
BMI, mean ± SD, kg/m^2^ (range)	18.22 ± 0.41 (14.31–24.65)
Age at diagnosis, *n* (%)	
A1 (≤ 16 years)	1 (2.5%)
A2 (17–40 years)	27 (67.5%)
A3 (> 40 years)	12 (30.0%)
Disease location, *n* (%)	
L1 (ileal)	24 (60.0%)
L2 (colonic)	4 (10.0%)
L3 (ileocolonic)	12 (30.0%)
Disease location, *n* (%)	
L1 (ileal)	24 (60.0%)
L2 (colonic)	4 (10.0%)
L3 (ileocolonic)	12 (30.0%)
Behaviors, *n* (%)	
B1 (non-stricturing, non-penetrating)	3 (7.5%)
B2 (stricturing)	16 (40.0%)
B3 (penetrating)	21 (52.5%)
P (perianal disease), *n* (%)	9 (22.5%)
Internal fistula or abscess, *n* (%)	13 (32.5%)

*BMI *Body mass index

In the control group, there were 21 males and 19 females, with an average age of 38.45 ± 1.08 (24–47) years and an average BMI of 18.68 ± 0.19 (16.44–20.62) kg/m^2^. There were no statistically significant differences in age, gender distribution, and BMI between the creeping fat group and the control group (*p* > 0.05).

### Measurements of dual-energy parameters

There was good inter-individual consistency in the measurements between the two radiologists, with intra-class correlation coefficients ranging from 0.84 to 0.93 (95% CI = 0.70, 0.96), and the measurement data from either radiologist was randomly selected for analysis.

As shown in Table 2, λ_HU_, NFIC, NFWC, and NFVF at the enteric phase were significantly different between MAT in the controls and creeping fat in the CD group (Kruskal–Wallis *H* test: *p* < 0.001; Wilcoxon test: *p* < 0.001).

**Table 2:** Comparison of spectral quantitative parameters between mesenteric adipose tissue in the controls, “normal” mesenteric adipose tissue with Crohn’s disease, and creeping fat with Crohn’s disease

Quantitative parameter	Controls (*n* = 40)	“Normal” MAT with CD (*n* = 40)	Creeping fat with CD (*n* = 40)	*p* value*	*p* value**	*p* value***
λ_HU_ (HU/keV) at the enteric phase	− 1.33 (− 1.58 to − 1.24)	− 1.22 (− 1.40 to − 1.00)	0.58 (− 0.05 to 1.20)	< 0.001	< 0.001	0.135
NFIC at the enteric phase	1.00 (1.00–1.01)	1.00 (1.00–1.005)	1.05 (1.02–1.07)	< 0.001	< 0.001	1.000
NFWC at the enteric phase	0.83 (0.72–0.94)	0.89 (0.76–0.99)	− 0.41 (− 0.92 to − 0.46)	< 0.001	< 0.001	1.000
NFVF at the enteric phase	1.00 (0.99–1.02)	1.00 (0.98–1.08)	0.33 (0.26–0.79)	< 0.001	< 0.001	1.000

^*^Comparisons between “normal” MAT with CD and creeping fat with CD

^**^Comparisons between MAT in the controls and creeping fat with CD

^***^Comparisons between MAT in the controls and “normal” MAT with CD

*CD*, Crohn’s disease; *λ*_*HU*_, slope of the spectral Hounsfield unit curve; *MAT*, mesenteric adipose tissue; *NFIC*, normalised fat-iodine concentration; *NFWC*, normalised fat–water concentration; *HU*, Hounsfield unit; *NFVF*, normalised fat volume fraction

According to Table 3, λ_HU_, NFIC, NFWC, and NFVF at the enteric phase demonstrated a significant difference between inactive and active CD (Mann–Whitney *U* test: *p* < 0.01).

**Table 3:** Spectral quantitative parameters of creeping fat with inactive and active Crohn’s disease

Quantitative parameter	Inactive CD (*n* = 14)	Active CD (*n* = 26)	*p* value*
λ_HU_ (HU/keV) at the enteric phase	− 0.28 (− 0.73 to − 0.11)	1.00 (0.53–1.57)	< 0.001
NFIC at the enteric phase	1.02 (1.00–1.04)	1.07 (1.04–1.08)	< 0.001
NFWC at the enteric phase	0.52 (0.14–1.00)	− 0.77 (− 0.97 to − 0.35)	< 0.001
NFVF at the enteric phase	0.79 (0.67–0.92)	0.29 (0.20–0.38)	< 0.001

^*^Mann–Whitney *U* test

*λ*_*HU*_, slope of the spectral Hounsfield unit curve; *NFIC*, normalised fat-iodine concentration; *NFWC*, normalised fat–water concentration; *HU*, Hounsfield unit; *NFVF*, normalised fat volume fraction

ROC curve analysis showed that the AUC of λ_HU_ at the enteric phase was highest for the differential diagnosis of creeping fat between inactive and active CD (Table 4).

**Table 4:** Receiver operating characteristic curves of quantitative parameters for the differential diagnosis of creeping fat with inactive and active Crohn’s disease

Quantitative parameter	AUC	Threshold	Sensitivity (%)	Specificity (%)
λ_HU_ (HU/keV) at the enteric phase	0.93 (0.81–0.99)	0.20	92.31 (74.9–99.1)	85.71 (57.2–98.2)
NFIC at the enteric phase	0.84 (0.69–0.94)	1.05	69.23 (48.2–85.7)	92.86 (66.1–99.8)
NFWC at the enteric phase	0.92 (0.79–0.98)	–0.13	92.31 (74.9–99.1)	85.71 (57.2–98.2)
NFVF at the enteric phase	0.86 (0.71–0.95)	0.59	84.62 (65.1–95.6)	78.57 (49.2–95.3)

*λ*_*HU*_, slope of the spectral Hounsfield unit curve; *NFIC*, normalised fat-iodine concentration; *NFWC*, normalised fat–water concentration; *HU*, Hounsfield unit; *NFVF*, normalised fat volume fraction; *AUC*, area under the curve

### Assessment of radiation dose

The mean volume CT dose index (CTDI_vol_) and dose-length product (DLP) were 10.10 mGy and 548.29 ± 6.37 (482.56–749.50) mGy·cm at the enteric phase, respectively.

## Discussion

The pathologic changes of the mesentery in CD are commonly seen at the sites of affected bowels and are closely related to the stage of disease, the activity of inflammation, and complications. In chronic advanced patients, the affected mesentery usually shows chronic inflammation, becomes thicker and tends to wrap the diseased bowel, and is recognised as creeping fat. In creeping fat, the MAT is hypertrophy with increased and disordered small round adipocytes, increased collagen content, and thickened, proliferated mesenteric vascular walls with stenotic or occluded vessels [7, 21]. With severe pathological changes, creeping fat may hinder the favourable treatment response of drugs and become an indication for surgery. To accurately assess creeping fat, this study adopted a surgery patient cohort and built a clinical-surgical, radiological, and histopathological correlation. In our study, by implementing the enteric phase spectral CTE scan, we obtained a series of new quantitative parameters for creeping fat with an imaging-surgical correlation in CD. Our study showed that λ_HU_, NFIC, NFWC, and NFVF at the enteric phase were significantly different among MAT in the controls, “normal” MAT with CD, and creeping fat with CD, while there was no significant difference between MAT in the controls and “normal” MAT with CD. This suggests that dual-energy CT can readily discriminate the abnormal MAT from the normal MAT. The HU curves of creeping fat with CD are inverted compared to those of MAT in the controls and “normal” MAT in CD. λ_HU_ reflects the slope of the HU curve for the X-ray attenuation coefficient of different tissues as the X-ray energy level changes from 40 to 140 keV [31], which could be helpful in identifying different components in the tissues [32, 33]. Our study has shown that the λ_HU_ of creeping fat is 0.58 at the enteric phase while the λ_HU_ of “normal” MAT with CD is − 1.22 at the enteric phase. The results indicate that the components in the creeping fat tissues are significantly different from those in the “normal” MAT. With material decomposition tools for quantifying fat, water, and iodine, NFIC of creeping fat was higher but NFWC and NFWC were lower than MAT in the controls or “normal” MAT with CD at the enteric phase. These findings are corresponding to the pathologic changes of creeping fat. The clinical pathology [34] and the results of animal models [35] both have shown that creeping fat is an inflammatory adipose tissue with fibrofatty proliferation [7], rather than a typical fat tissue. The creeping fat contains more micro-vessels and other non-fat components (e.g. oedema, collagen, and fibrous tissue) [35], contributing to the increased NFIC and decreased NFWC and NFWC.

Our study showed that λ_HU_, NFIC, NFWC, and NFVF of creeping fat at the enteric phase were significantly different between inactive CD and active CD (all *p* < 0.01). Creeping fat with active diseases is oedematous and congested, so it contains more water than inactive diseases. On the contrast-enhanced images, it has a higher concentration of iodinated contrast medium for active diseases than inactive diseases. These results suggest that the dual-energy parameters of creeping fat are useful in quantitatively evaluating the activity of CD. Since these parameters are obtained noninvasively, they can be used to monitor disease progression.

The ROC analysis results have shown that the quantitative parameters (λ_HU_, NFIC, NFWC, and NFVF) at the enteric phase are efficient in the discrimination between creeping fat with inactive and active CD, among which the λ_HU_ at the enteric phase had the highest diagnostic accuracy (AUC = 0.93; 95% CI, 0.81–0.99; *p* < 0.001).

Mahmood et al. [36] found that radiation dose, particularly organ dose, was lower with rapid-switching dual-energy CT (rsDECT) compared with conventional single-energy computed tomography CT (SECT), especially in smaller-sized patients. The organ dose was, on average, 37.4% less with rapid-switching dual-energy CT. Our dual-energy CTE scanning protocol showed relatively lower values of CTDI_vol_ and DLP (10.10 mGy and 548.29 ± 6.37 mGy·cm) at the enteric phase, as compared to the low-dose CTE protocol (with CTDI_vol_ and DLP of 12.29 ± 3.33 mGy and 604.98 ± 180.59 mGy·cm) reported by Ippolito et al. [37]. Low-dose dual-energy CTE scanning protocol therefore can be considered a useful tool in the management of CD patients, considering the young age of patients and the frequent imaging follow-up required [37].

Higher visceral adipose tissue with CD was associated with more hospitalisations, increased complicated disease, shorter intervals from diagnosis to surgery, and higher disease activity scores [38]. Visceral adipose tissue accumulation was a prospective risk factor for increased disease activity in CD [39]. A high visceral adipose tissue value was associated with postoperative recurrence of CD [40]. Alteration of body composition (subcutaneous and visceral adipose tissue) appears to be a marker of disease severity and complex phenotypes [41, 42]. Because of the important role mesentery plays in the disease development of CD, the qualitative evaluation of creeping fat may be beneficial to the clinical management of CD. Recently, Coffey et al. [5] and Li et al. [16] have developed a mesenteric disease activity index to quantify the severity of CD according to the grade of mesenteric thickening and fat wrapping. However, this index is based on the morphologic findings and may be difficult to measure on the axial images. Our study may provide an alternative simple method in quantitatively evaluating the activity of the mesentery of CD.

This study has several limitations. Firstly, it was a single-centre clinical study, and the sample size of creeping fat with surgical resection was limited. However, all the investigations in this study were performed using the same CT scanner and nearly all the patients were operated by a single surgeon who is experienced in the surgical management of CD. This helped to keep the diagnostic criteria and measurement consistent. Secondly, subjects with different subtypes (such as terminal ileal, colonic, and ileocolonic subtypes) of CD were included in our study, and future prospective studies focused on the potential pathological alterations underlying the creeping fat on the CTE images in each CD subtype may be attempted. Thirdly, the follow-up patients with CD can be further followed to study the relationship between the quantitative parameters of MAT and creeping fat in dual-energy CTE and the disease outcome, treatment response, or prognosis, and even compare the parameter changes before and after treatment.

In conclusion, dual-energy CTE with a series of spectral parameters can accurately distinguish normal MAT and creeping fat. Spectral parameters probably help quantitatively evaluate the disease activity of creeping fat in CD. Dual-energy CTE may benefit the clinical decision and surveillance of CD.

## Data Availability

All data generated or analysed during this study are included in this published article.

## Abbreviations

AUC: Area under the curve
BMI: Body mass index
CD: Crohn’s disease
CDAI: Crohn’s disease activity index
CTDI_vol_: Volume CT dose index
CTE: CT enterography
DLP: Dose-length product
FVF: Fat volume fraction
GSI: Gemstone spectral imaging
MAT: Mesenteric adipose tissue
MMD: Multi-material decomposition
NFIC: Normalised fat–water concentration
NFVF: Normalised fat volume fraction,
NFWC: Normalised fat-iodine concentration
ROC: Receiver operating characteristic curve
ROI: Region of interest
VMC: Virtual monochromatic
λ_HU_: The slope of the Hounsfield unit curve
